# Non-progressive CRMP-5-Associated Perifoveal Retinopathy in a Breast Cancer Patient: A Rare Paraneoplastic Phenomenon

**DOI:** 10.7759/cureus.74342

**Published:** 2024-11-24

**Authors:** Isabella Canut, Rachel Bielling, Fiona Steinmetz, Raquel Esquivel, Charles Maitland

**Affiliations:** 1 Neurology, Philadelphia College of Osteopathic Medicine, Suwanee, USA; 2 Neurology, Florida State University College of Medicine, Tallahassee, USA

**Keywords:** breast cancer, crmp-5 antibodies, paraneoplastic retinopathy, paraneoplastic syndromes, photophobia

## Abstract

Paraneoplastic retinopathy (PR) is a rare autoimmune condition typically associated with progressive visual loss and is often linked to anti-recoverin antibodies. Paraneoplastic optic neuropathy (PON) is classically associated with collapsin response-mediator protein (CRMP-5). We present a unique case of non-progressive CRMP-5-associated perifoveal retinitis in a 79-year-old female with a history of breast carcinoma, who has maintained a stable visual acuity over an extended follow-up period of three years.

## Introduction

Paraneoplastic syndromes, derived from the Greek prefix 'para' meaning 'besides', and 'neoplasm' referring to a tumor, encompasses a group of rare disorders that occur due to an underlying malignancy. These syndromes are characterized by complex clinical pictures resulting from the immune system's response to an occult or known cancer, which leads to a variety of systemic manifestations. Paraneoplastic syndromes commonly manifest in patients with breast cancer, lung cancer, hematological malignancies, medullary thyroid cancer, gynecological malignancies, and prostate cancer [1]. It is noteworthy that these syndromes affect less than 10% of patients with cancer, with visual system involvement in less than .01% of cases [2].

Paraneoplastic retinopathy (PR) presents with a gradual, painless loss of vision and photophobia [3]. PR is associated with the presence of recoverin, a retinal antigen, and is typically seen with cases of small cell carcinoma and melanoma. Paraneoplastic optic neuropathy (PON) represents another paraneoplastic syndrome causing visual impairment. PON typically presents as a subacute and progressive bilateral visual loss, associated with optic disc swelling [2]. Collapsin response-mediator protein-5 (CRMP-5) is linked to PON, however, its exact pathogenesis to immunogenicity is not known.

This article was previously presented as a meeting abstract at the 2022 North American Neuro-Ophthalmology Society meeting on February 13, 2022.

## Case presentation

A 79-year-old Caucasian female with a medical history of breast carcinoma in remission, previously treated with trastuzumab and anastrozole, presented with severe bilateral photophobia without noticeable visual loss. Her visual acuity was preserved at 20/20 bilaterally. She had intact pupillary light reflexes with no relative afferent pupillary defect. Extraocular movements were full in all directions, and there was no nystagmus. However, her color vision was notably absent during testing with a score of 0/6 bilaterally on the Hardy-Rand-Rittler (HRR) color-blind test. She denied any central or peripheral visual field defects or significant vision changes.

**Figure 1:**
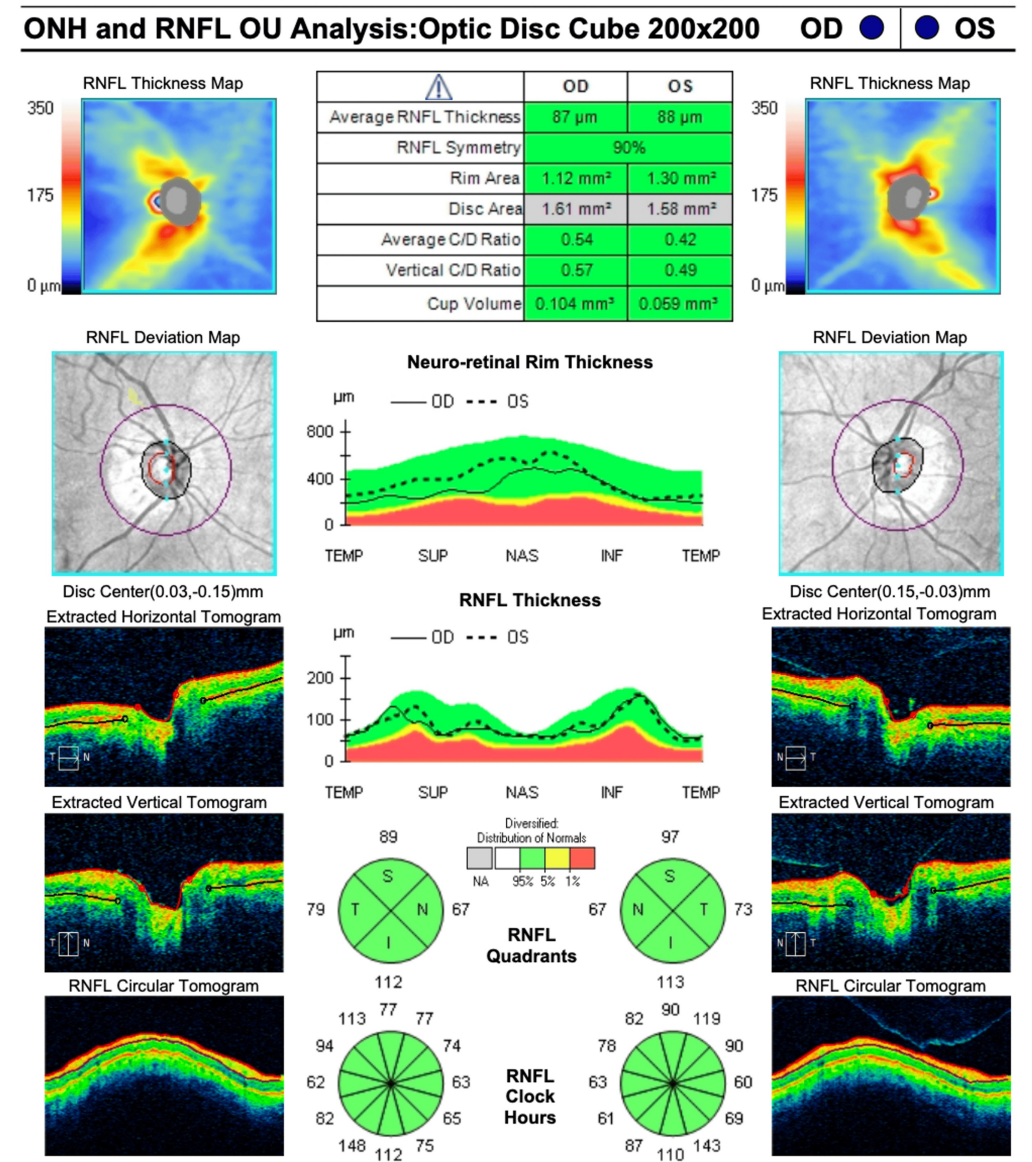
Optical coherence tomography (OCT) results demonstrating retinal nerve fiber layer (RNFL) and neuro-retinal rim analysis The OCT results show a normal RNFL in the right eye (OD) of 87 µm and 88 µm in the left eye (OS) and normal neuro-retinal rim thickness in both eyes. OU: oculus uterque (both eyes); MD: mean deviation

**Figure 2:**
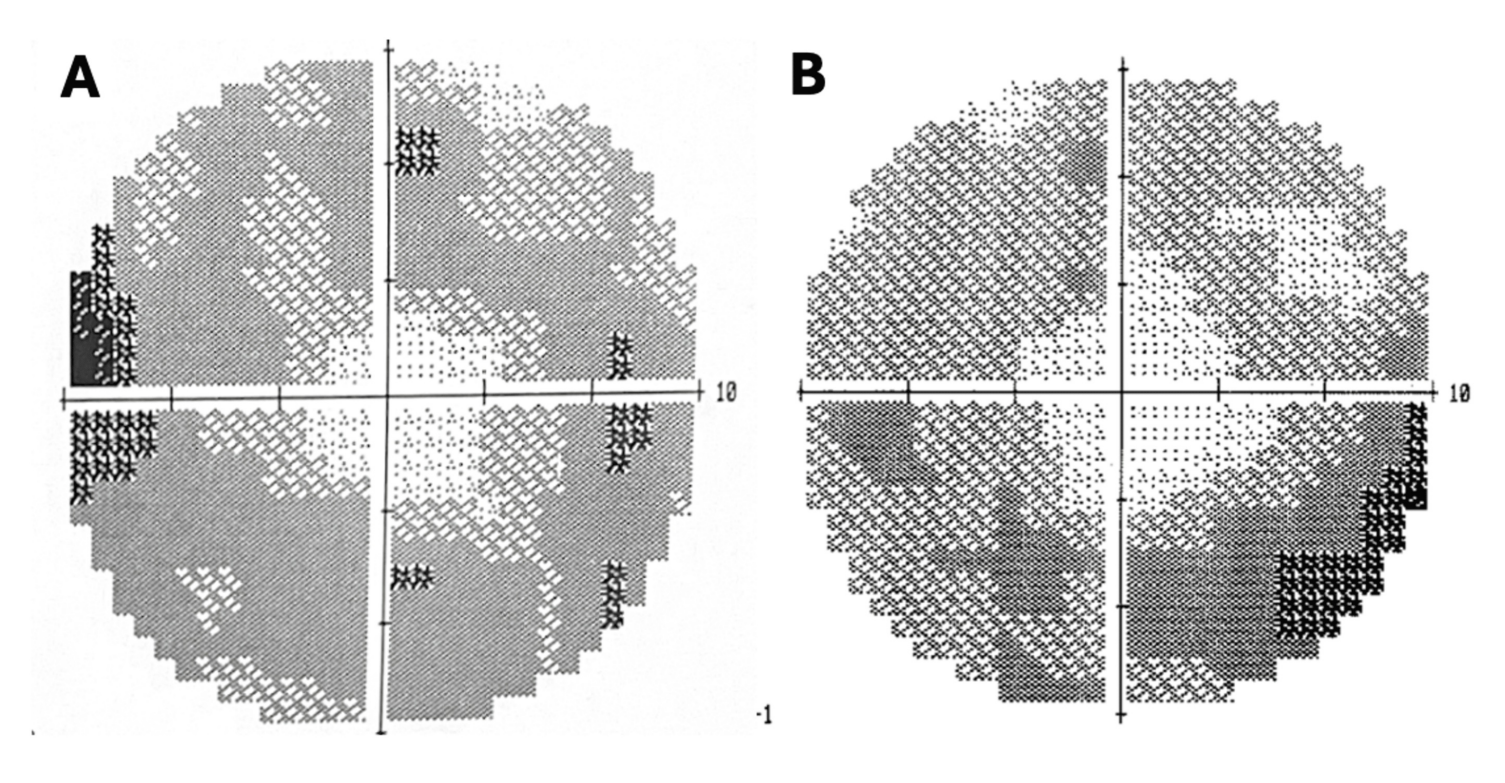
Humphrey's 10-2 visual fields Visual fields showing a bilateral ring scotoma with sparing of the foveal area (A, B). The image on the left (A) is the left eye with an MD score of -17.04 and the right eye (B) has an MD score of -14.28. MD: mean deviation

A detailed fundic examination, including optical coherence tomography (OCT), revealed normal nerve fiber layer thickness but significant depletion of the ganglion cell layer (Figure 1). Humphrey’s 10-2 visual field testing demonstrated bilateral central sparing with the presence of peripheral ring scotomas (Figure 2). A neurological examination was otherwise unremarkable, with normal strength, reflexes, sensation, and coordination in all extremities. Magnetic resonance imaging (MRI) of the brain and orbits was unremarkable, ruling out any mass or structural lesion.

**Figure 3:**
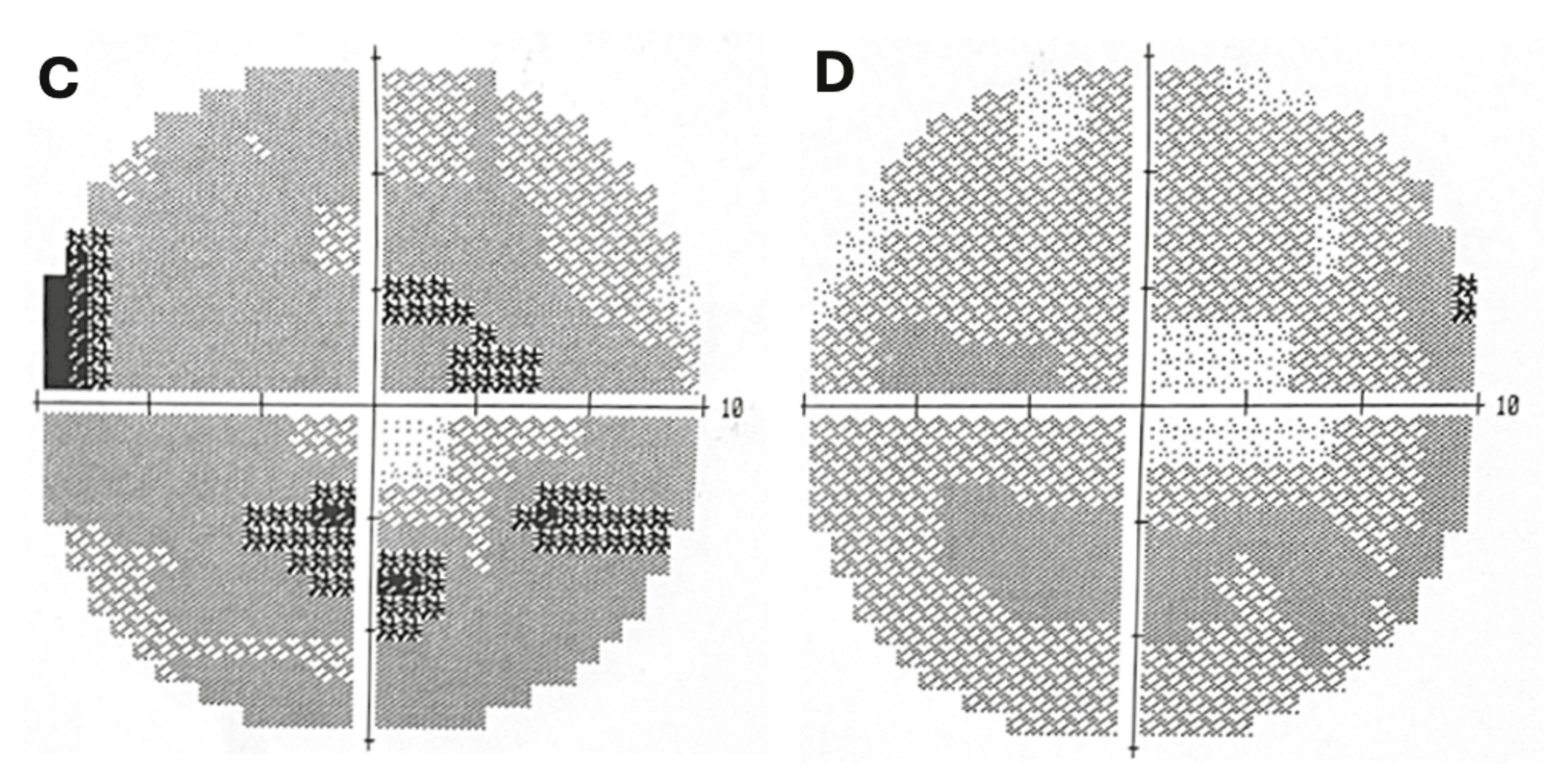
Humphrey's 10-2 visual fields Repeat visual fields taken three years later showing a bilateral ring scotoma with sparring of the foveal area (C, D). The image on the left (C) is the left eye with an MD score of -17.96 and the right eye (C) has an MD score of -14.29. MD: mean deviation

Western blot testing for CRMP-5 initially tested positive, then negative when repeated seven months later. Interestingly, the repeated labs also revealed anti-retinal antibodies targeting 27 kDa, 31 kDa, and 180 kDa proteins. Despite these findings, the patient’s visual acuity and visual fields have remained stable throughout a three-year follow-up period, seen in repeat visual fields (Figure 3), without any evidence of progressive visual loss.

## Discussion

PR is linked to retinal antigens such as recoverin. PR typically presents with a gradual, painless visual loss over a period of weeks to months, along with photosensitivity and visual field deficits [3]. Retinal antigens lead to progressive retinal degeneration with cone and rod photoreceptor dysfunction. Cone dysfunction manifests as photosensitivity, photopsia, and decreased central vision. Rod dysfunction involves nyctalopia (night blindness), ring scotoma, or other peripheral vision loss. The most specific and sensitive antigen associated with paraneoplastic retinopathy is recoverin, a 23-kDa protein involved in the activation and recovery of guanylate cyclase, which influences rhodopsin function [4]. Inactivation of the recoverin protein results in the loss of photoreceptors. Recoverin-related retinopathy is commonly associated with small-cell lung carcinoma and the diagnosis is commonly reported prior to the diagnosis of cancer; therefore, when suspected, screening for an underlying tumor needs to be done [5]. Patients with a positive anti-recoverin antibody commonly have a more rapid onset of visual decline [3].

PON typically presents as a gradual visual loss that becomes bilateral within weeks to months and leads to severe visual loss. In addition to visual loss, other symptoms include blurred vision, phosphenes, tunnel vision, and visual field defects. The associated antibody with PON is CRMP-5, a 62-kDa protein involved in axon guidance and neuronal development. Patients with a positive CRMP-5 antibody may have other symptoms, including dementia, seizures, anosmia, ageusia, dysphagia, limb weakness, Parkinsonism, myoclonus, chorea, hemiballismus, dyskinesia, akathisia, ataxia, early satiety, constipation/diarrhea, dry mouth, nausea, and weight loss [5]. 

The Differential diagnosis for a patient presenting with a new onset of bilateral photophobia and abnormal visual field findings in a patient with a history of cancer includes several possibilities including autoimmune retinopathy, optic neuritis, paraneoplastic optic neuropathy, toxic or nutritional optic neuropathy, or primary retinal degeneration. Autoimmune retinopathy, specifically cancer-associated retinopathy (CAR) was a primary consideration. However, CAR is commonly associated with anti-recoverin antibodies and leads to a progression of visual loss. The absence of recoverin antibodies and the lack of visual loss argues against this diagnosis. Optic neuritis and paraneoplastic optic neuropathy were also considered, however, her unremarkable MRI, stable visual acuity, and visual acuity showing parafoveal retinopathy make these diagnoses unlikely. Conditions such as vitamin B12 deficiency or exposure to toxins were also considered. However, the patient’s normal serum B12 and folate levels in addition to the absence of known toxin exposures helped rule out these possibilities. The patient’s absence of progressive visual loss in addition to her history of breast carcinoma made it unlikely that this occurred due to conditions such as retinitis pigmentosa or other hereditary retinal dystrophies. The presence of CRMP-5 antibodies and anti-retinal antibodies, combined with the lack of progressive visual loss, pointed toward a diagnosis of paraneoplastic retinopathy.

There is no standardized treatment protocol for paraneoplastic retinopathy. Treatment of the underlying cancer alone does not prevent visual decline. Treatment includes long-term immunosuppression with or without local steroids [6]. 

It is uncommon to observe CRMP-5 in cases of paraneoplastic retinopathy with perifoveal involvement. The presence of anti-retinal antibodies, identified later in the disease course, suggests a possible evolution of the immune response from the optic nerve to retinal structures. The patient’s stable visual function over time raises questions about the underlying mechanisms of this non-progressive form of paraneoplastic retinopathy. Given that most forms of paraneoplastic retinopathy tend to be progressive, this case may suggest that early detection and immune modulation may play a role in preventing further retinal degeneration. In addition, the transient presence of CRMP-5 antibodies may indicate a fluctuating immune activity, possibly driven by the patient’s underlying breast cancer or its treatment. The patient’s visual disturbances, including severe photophobia and color vision loss, were associated with ganglion cell depletion and peripheral ring scotomas, but central vision remained intact.

## Conclusions

To date, the paraneoplastic retinopathy appears stable and non-progressive. It is plausible that the initial development of retinitis with anti-optic nerve antibodies, followed by the emergence of anti-retinal antibodies, was due to cancerous activity. Reports of non-progressive paraneoplastic retinopathy are rare. The relationship between CRMP-5 antibodies, optic neuropathy, and retinopathy warrants further investigation, especially in cases of breast cancer. This case’s presentation of stable retinopathy with sparing of the optic nerve and retina may suggest an expansion of the spectrum of CRMP-5-related paraneoplastic syndromes. The role of CRMP-5 antibodies in this atypical presentation may alert clinicians to consider paraneoplastic retinopathy in similar cases of unexplained photophobia or visual disturbances. The case also emphasizes the importance of ongoing monitoring in patients with a history of cancer, even in patients whose conditions appear stable.

## References

[1] Thapa B, Mahendraker N, Ramphul K (2024). Paraneoplastic Syndromes. https://www.ncbi.nlm.nih.gov/books/NBK507890/.

[2] Sorrentino DM, Lee J, Bonhomme GR (2019). CRMP-5 positive paraneoplastic optic neuropathy as initial presentation of small cell lung cancer. Clin Oncol.

[3] Rahimy E, Sarraf D (2013). Paraneoplastic and non-paraneoplastic retinopathy and optic neuropathy: evaluation and management. Surv Ophthalmol.

[4] C E Thirkill, R C Tait, N K Tyler (1992). The cancer-associated retinopathy antigen is a recoverin-like protein. Invest Ophthalmol Vis Sci.

[5] Hickman SJ (2022). Paraneoplastic syndromes in neuro-ophthalmology. Ann Indian Acad Neurol.

[6] Thomas A (2024). Paraneoplastic retinopathies: an update on pathogenesis, diagnosis and management. Ann Eye Sci.

